# Long-Term Survival and Induction of Operational Tolerance to Murine Islet Allografts by Co-Transplanting Cyclosporine A Microparticles and CTLA4-Ig

**DOI:** 10.3390/pharmaceutics15092201

**Published:** 2023-08-25

**Authors:** Purushothaman Kuppan, Jordan Wong, Sandra Kelly, Jiaxin Lin, Jessica Worton, Chelsea Castro, Joy Paramor, Karen Seeberger, Nerea Cuesta-Gomez, Colin C. Anderson, Gregory S. Korbutt, Andrew R. Pepper

**Affiliations:** 1 Alberta Diabetes Institute, University of Alberta, Edmonton, AL T6G 2E1, Canada; kuppan@ualberta.ca (P.K.); jmwong3@ualberta.ca (J.W.); sankelly@ualberta.ca (S.K.); jlin9@ualberta.ca (J.L.); jldesaul@ualberta.ca (J.W.); ccastro@ualberta.ca (C.C.); jparamor@ualberta.ca (J.P.); ks1@ualberta.ca (K.S.); cuestago@ualberta.ca (N.C.-G.); colinand@ualberta.ca (C.C.A.); 2 Department of Surgery, University of Alberta, Edmonton, AL T6G 2E1, Canada

**Keywords:** type 1 diabetes, localized drug delivery, cyclosporine A, murine islet allograft, islet transplantation, CTLA4-Ig

## Abstract

One strategy to prevent islet rejection is to create a favorable immune-protective local environment at the transplant site. Herein, we utilize localized cyclosporine A (CsA) delivery to islet grafts via poly(lactic-co-glycolic acid) (PLGA) microparticles to attenuate allograft rejection. CsA-eluting PLGA microparticles were prepared using a single emulsion (oil-in-water) solvent evaporation technique. CsA microparticles alone significantly delayed islet allograft rejection compared to islets alone (*p* < 0.05). Over 50% (6/11) of recipients receiving CsA microparticles and short-term cytotoxic T lymphocyte-associated antigen 4-Ig (CTLA4-Ig) therapy displayed prolonged allograft survival for 214 days, compared to 25% (2/8) receiving CTLA4-Ig alone. CsA microparticles alone and CsA microparticles + CTLA4-Ig islet allografts exhibited reduced T-cell (CD4^+^ and CD8^+^ cells, *p* < 0.001) and macrophage (CD68^+^ cells, *p* < 0.001) infiltration compared to islets alone. We observed the reduced mRNA expression of proinflammatory cytokines (*IL-6*, *IL-10*, *INF-γ*, and *TNF-α*; *p* < 0.05) and chemokines (*CCL2*, *CCL5*, *CCL22*, and *CXCL10*; *p* < 0.05) in CsA microparticles + CTLA4-Ig allografts compared to islets alone. Long-term islet allografts contained insulin^+^ and intra-graft FoxP3^+^ T regulatory cells. The rapid rejection of third-party skin grafts (C3H) in islet allograft recipients suggests that CsA microparticles + CTLA4-Ig therapy induced operational tolerance. This study demonstrates that localized CsA drug delivery plus short-course systemic immunosuppression promotes an immune protective transplant niche for allogeneic islets.

## 1. Introduction

Islet transplantation is a proven strategy to restore glycemic control, reduce hypoglycemic unawareness, and stabilize HbA1c levels for a subset of patients with type 1 diabetes (T1D) [1]. A long-term follow-up study has shown that graft survival, based on C-peptide concentrations (<0.1 nmol/L), was 48% at 20 years after the first transplantation [2]. Even though marked improvements have been made in clinical islet transplantation, the reliance on chronic immunosuppression to protect islets from host auto- and alloreactivity remains a significant obstacle to patient inclusion. These obligatory and often diabetogenic drugs pose inherit potential risks, including cytotoxic effects on vital organs, severe infections and tumorigenesis [3,4]. To reduce systemic side effects, targeted strategies such as the islet graft localized delivery of immunosuppressive agents is an attractive strategy to abrogate drug toxicity and improve β-cell survival and a possible adjuvant to achieving operational (islet-graft-specific) tolerance. Herein, we have delivered a subtherapeutic dose of CsA locally to the graft in combination with short-term systemic CTLA4-Ig to reduce inflammation and improve islet survival and engraftment.

CsA, a calcineurin inhibitor, is a potent immunosuppressive drug used to prevent allograft rejection in solid organ transplantation and to treat autoimmune diseases [5]. In islet transplantation, CsA has been administered systemically along with immune cell-depleting agents (Anti-Thymocyte Globulin (ATG), Alemtuzumab) to prevent islet graft rejection as it blocks the interleukin (IL)-2–dependent proliferation and differentiation of T cells [6]. Additionally, CsA binds with cyclophilin D, which prevents mitochondrial permeability transition pores opening under stress conditions, stabilizing mitochondrial functions and preventing cell death [7]. However, it is well documented that high doses of CsA impair β-cell function [8,9,10,11,12] and that in nephrotoxicity is a serious side effect that limits CsA’s widespread clinical application [13]. While systemically administered CsA dosage between 1 and 50 mg/kg results in modest islet murine allograft protection (~1 to 2 weeks survival) [6,14,15], it also induces cytotoxicity and islet cell damage [16]. Additionally, Arita et al. found that at a systemic CsA dose of 30 mg/kg resulted in morbidity and mortality despite having functioning murine islet allografts [14].

Herein, poly(lactic-co-glycolic acid) (PLGA) microparticles were used for the localized release of cyclosporine A (CsA) to allogeneic murine islet grafts. PLGA is a Food and Drug Administration-authorized biodegradable and biocompatible polymer approved for numerous biomedical applications, including drug delivery [17]. Our group has recently shown that the co-localization of dexamethasone (Dex)-eluting PLGA microparticles to islet allografts with CTLA4-Ig therapy rendered 80% of the recipients euglycemic for 60 days posttransplant compared to 40% in recipients treated with empty microparticles + CTLA4-Ig [18]. Although the delivery of CsA using PLGA microparticles has been previously utilized [19,20], the targeted and localized effect of CsA on islet transplantation remains unexplored. Therefore, we sought to investigate the ability of a localized subtherapeutic dose of CsA delivered via PLGA microparticles to improve islet allograft function and circumvent complications associated with systemic immunosuppression. The local release of CsA is favored over systemic administration as a means to offset the harmful or adverse immune-dampening effects of systemic administration. Here, we described the optimization of CsA-eluting PLGA microparticle fabrication and in vitro characterization. Next, we assessed the potential adverse effects of CsA-eluting PLGA microparticles using a syngeneic transplant model. Subsequently, we examined the ability of CsA microparticles to enhance murine islet allograft function in a fully mismatched model (minor and major histocompatibility complex–MHC). Finally, we examined the ability of another clinically relevant immunosuppressant, CTLA4-Ig, to work in concert with CsA microparticle co-delivery to improve alloislet engraftment and survival.

## 2. Materials and Methods

### 2.1. Preparation and Characterization of CSA-Eluting PLGA Microparticles

The procedure for the preparation and characterization of CSA-eluting PLGA microparticles can be found in Supplementary Material.

### 2.2. In Vivo Drug Release Characterization

In vivo CsA release study was conducted in nondiabetic B6.129S7-Rag1^tm1Mom^/J mice (Jackson Laboratory, Bar Harbor, ME, USA) following implantation of 4 mg of CsA microparticles mixed with collagen type I (Corning, NY, USA) and implanted under the kidney capsule (KC). At 0, 1, 3, 7, 14, and 21 days post-implantation (*n* = 4 mice/time pt), microparticle-bearing kidneys were removed, homogenized, and analyzed for CsA using HPLC (Agilent Technologies, 1200 series, Santa Clara, CA, USA) [18,21]. Daily in vivo release of CsA was calculated.

### 2.3. Islet Isolation, Transplantation, and Metabolic Follow-Up

Procedures for islet isolation, transplantation and metabolic follow-up can be found in Supplementary Material. Syngeneic islet transplantation studies (BALB/c to BALB/c) were conducted to assess the adverse effect of CsA microparticles; subsequently, allogeneic islet transplants (BALB/c to C57BL/6) were conducted. Transplants consisted of 500 islets combined with 4 mg CsA microparticles under the KC of diabetic mice. Details of all experimental groups can be found in Supplementary Material.

### 2.4. Intra-Islet Graft Proinflammatory Cytokine Analysis and Gene Expression Analysis

Acute allotransplant studies were conducted in which diabetic C57BL/6 mice received 500 BALB/c islets + 4 mg CsA microparticles (*n* = 3) or 500 BALB/c islets + 4 mg CsA microparticles + CTLA4-Ig (10 mg/kg, day 0, 2, 4, and 6 posttransplant). After 7 days, grafts were removed and analyzed for proinflammatory cytokines and gene expression. Procedures for intra-islet graft proinflammatory cytokine analysis and gene expression analysis and details of all experimental groups can be found in Supplementary Material.

### 2.5. Islet Graft Immunohistochemical Analysis

At different time points post-transplant, islet grafts were fixed in formalin, processed, and sections were immuno-histochemically stained. Histological procedures can be found in Supplementary Material.

### 2.6. Assessment of Tolerance Induction by Allogeneic Skin Graft Transplantation

The procedure for the assessment of tolerance induction by allogeneic skin graft transplantation can be found in Supplementary Material. Briefly, skin grafts were conducted on long-term functioning islet allograft recipients, to investigate the possibly of the induction of transplant tolerance [22,23].

### 2.7. Statistical Analysis

All data are presented as mean ± standard error mean (SEM). Statistical significance between the treatment groups was calculated by one-way ANOVA (analysis of variances) and unpaired *t*-test. A Tukey posthoc test was used to analyze variances for multiple comparisons between the study groups. The Kaplan–Meyer survival function of alloislet transplants and skin transplants was compared using the log-rank (Mantel–Cox) statistical analysis. A 95% confidence interval was used as a threshold for significance, *p* < 0.05.

## 3. Results

### 3.1. CsA Microparticle Preparation and Characterization

**Figure 1:**
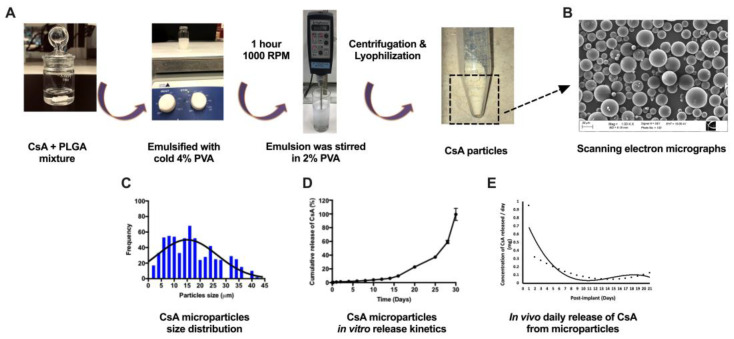
Schematic overview of the fabrication process for preparing cyclosporine A (CsA)-eluting poly(lactide-co-glycolic acid) (PLGA) microparticles via a single emulsion (O/W) solvent evaporation technique (**A**). PLGA and CsA were dissolved in dichloromethane (DCM) followed by emulsified with cold polyvinyl alcohol (PVA). A scanning electron micrograph illustrates the surface morphology of CsA-loaded PLGA microparticles (**B**), and the histogram represents the size distribution of CsA particles (**C**). In vitro drug release characterization demonstrating the cumulative CsA release percentage (%) from PLGA-CsA particles over 30 days (*n* = 4) (**D**). The daily released concentration of CsA was extrapolated from the in vivo cumulative release graphs with polynomial 3rd-order equations (**E**). Insert black line in Figure 1C and 1E represent the normal distribution. Data are expressed as mean ± SEM. Scale bars represent 20 μm for the scanning electron micrographs.

CsA-eluting PLGA microparticles were synthesized using a single emulsion solvent evaporation technique (Figure 1A). Scanning electron micrographs revealed that CsA-loaded microparticles were smooth and spherical in shape (Figure 1B). The size distribution of the CsA-loaded particles exhibited an average size of 16.3 ± 2.3 μm (Figure 1C). The total amount of CsA contained in the 10 mg of lyophilized PLGA CsA microparticles was 890.56 ± 14.61 μg and the encapsulation efficiency was 89.02 ± 1.46%. In vitro CsA microparticle release kinetics demonstrated that 100% of CsA was completely released from the particles within 30 days (Figure 1D). Measurement of the in vivo release of CsA showed a rapid release on day 1, likely associated with surface-bound CsA followed by a sustained CsA release between days 2 and 21 (Figure 1E). The released CsA concentration was between 0.1 and 0.3 mg/kg between days 2 and 8; then, CsA concentration was further reduced between days 9 and 21 (0.04 to 0.1 mg/kg). This in vivo release kinetics study demonstrated that our targeted daily average CsA release dose (0.1 mg/kg) was 50 times less than the systemic therapeutic CsA delivery dose (5 mg/kg). Additionally, CsA release was more accelerated in vivo than in in vitro. CsA concentration in KC decreased over time compared to the initial loading (day 0); approximately 57.97 ± 3.58% of CsA had been released by day 21 in vivo (Figure S1A–C), whereas 22.70 ± 0.69% of CsA had been released by the same time point in vitro (Figure S1D).

### 3.2. CsA-Eluting PLGA Microparticles Are Non-Toxic to Murine Syngeneic Islet Grafts

**Figure 2:**
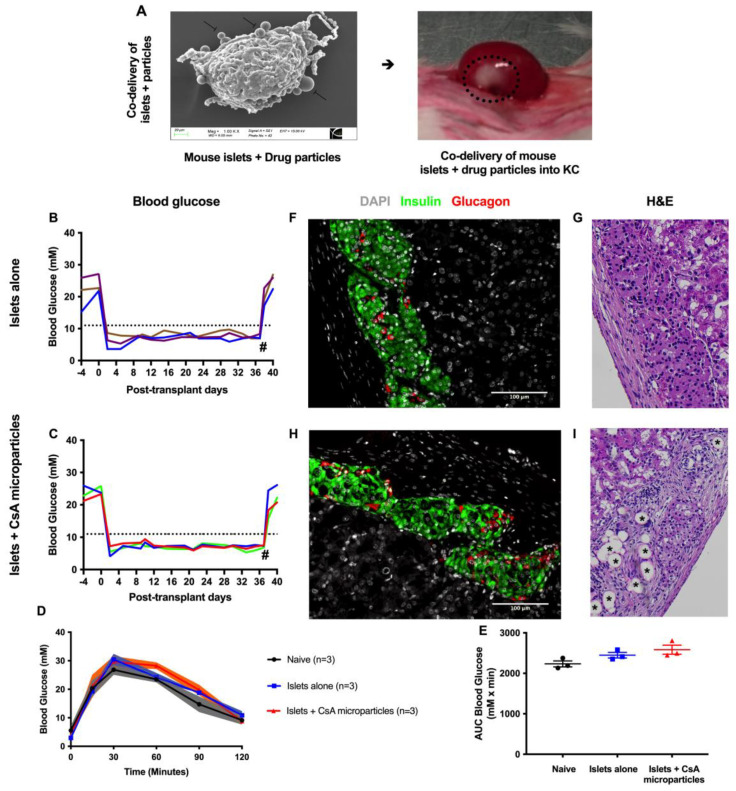
Syngeneic islet graft functional outcomes +/− CsA microparticle co-localization. Representative scanning electron micrographs of CsA microparticles shown to be attached to the islet surface (**A**). Dotted circle indicates the islet + drug particles in the KC. Diabetic BALB/c mice were transplanted with 500 BALB/c islets under the kidney capsule. Post-transplant blood glucose measurements of syngeneic islets alone (*n* = 3) (**B**) and syngeneic islets + CsA microparticles (*n* = 3) (**C**) recipients (colored lines indicate the individual mouse blood glucose). Hash (#) represents the time of nephrectomy of graft bearing kidney. Intraperitoneal glucose tolerance tests of syngeneic islet alone recipients (*n* = 3, blue) and syngeneic islets + CsA microparticles recipients (*n* = 3, red), at 35 days post-transplant. Naïve nondiabetic, nontransplant mice served as controls (*n* = 3, black). Mice were administered with 3 mg/g of 50% dextrose i.p. Blood glucose measurements were monitored at 0, 15, 30, 60, 90, and 120 min and analyzed for blood glucose (**D**) and area under the curve (**E**). Immunohistochemistry of syngeneic islet alone grafts (**F**,**G**) and syngeneic islets + CsA microparticles grafts (**H**,**I**). Green–insulin, red—glucagon, and grey—nuclei. Asterisks (*) represent microparticles that were co-localized to islet grafts under the KC. Data are expressed as mean ± SEM.

The adverse effects of localized CsA-eluting microparticles were examined using a syngeneic mouse model, whereby BALB/c islets were co-transplanted with 4 mg of CsA microparticles (*n* = 3) or islets alone (*n* = 3) into the KC of diabetic BALB/c mice. Scanning electron micrographs confirmed the co-localization of CsA-secreting microparticles on the surface of the islets (Figure 2A). All mice co-transplanted with syngeneic islets alone (*n* = 3, Figure 2B) or syngeneic islets + CsA microparticles (*n* = 3, Figure 2C) became euglycemic within 2 days and maintained euglycemia throughout the follow-up period. At 35 days post-transplant, CsA microparticles + syngeneic islet recipients demonstrated a similar glucose clearance profile compared to islet-alone recipients in response to a metabolic challenge (Figure 2D,E). These results confirmed that CsA microparticles did not impede islet engraftment or metabolic function at this dose. The graft-bearing kidneys were removed 35 days post-transplant, and subsequently all recipients promptly returned to a hyperglycemic state, confirming graft-dependent euglycemia. Intact islets were observed in both CsA microparticles + islet grafts and islet-alone grafts when stained for insulin and glucagon (Figure 2F–H) and hematoxylin and eosin (H & E) (Figure 2G–I). In addition, microparticles were observed as spherical vacuoles (indicated by *) within the CsA microparticles + islet grafts (Figure 2I), but not in the islet-alone grafts. These data demonstrate that 4 mg of lyophilized CsA microparticles (~356 μg of CsA) is non-toxic and does not compromise islet graft function.

### 3.3. Co-Localization of CsA-Eluting Microparticles Prolongs Murine Allograft Survival

**Figure 3:**
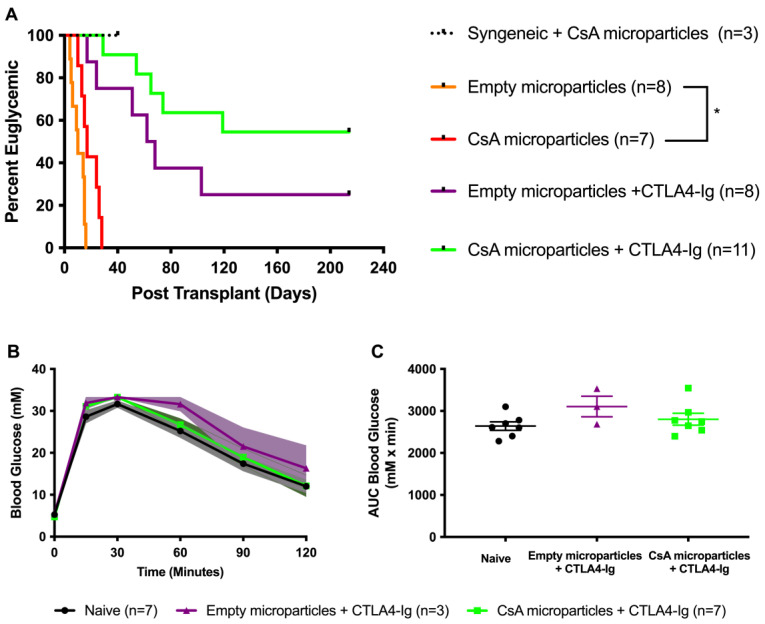
Islet allograft survival in mice transplanted with +/− CsA microparticles and +/− CTLA4-Ig (**A**). Syngeneic islet graft + CsA microparticles (*n* = 3, black) survival data were included as positive controls. Diabetic C57BL/6 mice were transplanted with 500 BALB/c islets under the KC. Islet allograft survival rates from recipients co-transplanted with islets + CsA-eluting microparticles (*n* = 7, red) significantly delayed the alloislet rejection compared to the islets + empty microparticles (*n* = 8, orange) (*p* < 0.05, Log-rank). Subsequently, two recipient groups (+/− CsA microparticles) were administered with CTLA4-Ig (10 mg/kg for short-term) i.p. at 0, 2, 4, and 6 days post-transplant. The recipients of empty microparticles + CTLA4-Ig (*n* = 8, purple) demonstrated significant graft survival compared to empty microparticles alone (*n* = 8, orange; *p* = 0.0001, log-rank). Similarly, recipients of CsA microparticles + CTLA4-Ig (*n* = 11, green) displayed significant graft survival compared to CsA-microparticles-alone recipients (*n* = 7, red; *p* < 0.0001, log-rank). The combination of CsA microparticles + CTLA4-Ig yielded a doubling in islet allograft survival, increasing survival by up to 214 days compared to empty microparticles + CTLA4-Ig (55 vs. 25%, respectively; *p* = 0.13, log-rank). Allograft recipients that maintained euglycemia at 214 days post-transplant were electively subjected to survival nephrectomy to confirm graft-dependent function. Intraperitoneal glucose tolerance tests were performed on allogeneic euglycemic recipients of CsA microparticles + CTLA4-Ig (*n* = 7, green) and empty microparticles + CTLA4-Ig (*n* = 3, purple) at 100 days post-transplant (**B**,**C**). Naïve nondiabetic non-transplanted mice served as controls (*n* = 7, black). Mice were administered with 3 mg/g of 50% dextrose i.p. Blood glucose measurements were monitored at 0, 15, 30, 60, 90 and 120 min and analyzed for blood glucose profiles (**B**) and area under the curve (**C**). Both CsA microparticles + CTLA4-Ig recipients (*n* = 7, green) and empty microparticles + CTLA4-Ig recipients (*n* = 3, purple) demonstrated a comparable glucose clearance to the naïve control (*n* = 7, black). Data are expressed as mean ± SEM. * *p* < 0.05.

Next, we conducted a series of fully allogeneic islet transplants (BALB/c islets (H2^d^) into diabetic C57BL/6 mice (H2^b^)) to study the immunomodulatory potential of the intragraft delivery of CsA-eluting PLGA microparticles. Islet allografts containing CsA-eluting microparticles (*n* = 7) survived significantly longer than islet allografts co-transplanted with empty microparticles (*n* = 8) (*p* < 0.05) (Figure 3A). Empty microparticles (control) recipients invariably lost graft function within 2 weeks posttransplant (median graft survival time (MST) = 9.87 ± 1.66 days). In contrast, CsA microparticle recipients showed significantly improved graft survival (MST = 19.00 ± 2.64 days). To determine whether CsA-eluting microparticles could promote long-term islet allograft survival when combined with short-course systemic immunosuppression, we subsequently conducted a series of allografts ± CsA microparticles, whereby the recipients were administered with a low dose of CTLA4-Ig (10 mg/kg) intraperitoneally (i.p.) at 0, 2, 4, and 6 days posttransplant. Recipients of CsA microparticles + CTLA4-Ig (*n* = 11) and empty microparticles + CTLA4-Ig (*n* = 8) displayed long-term allograft survival (Figure 3A). About 55% (6/11 recipients, MST = 147.70 ± 23.81 days) of the recipients co-transplanted with CsA microparticles + islets + CTLA4-Ig maintained allograft function for 214 days post-transplant; doubling the long-term euglycemia prevalence compared to CTLA4-Ig-alone monotherapy recipients (25% (2/8 recipients), MST = 94.13 ± 27.79 days). However, these data did not reach statistical significance (Figure 3A). Both alloislet recipients treated with CsA microparticles + CTLA4-Ig (*n* = 7) and alloislet recipients treated with empty microparticles + CTLA-4-Ig (*n* = 3) demonstrated a similar glucose clearance profile compared to naïve controls in response to a metabolic challenge (Figure 3B,C), confirming that co-localization ± CsA microparticles did not impede islet engraftment or metabolic function, at this dose.

### 3.4. Co-Localization of CsA Microparticles Modulates Intra-Islet Allograft Proinflammatory Responses and Immune Cell Infiltration

**Figure 4:**
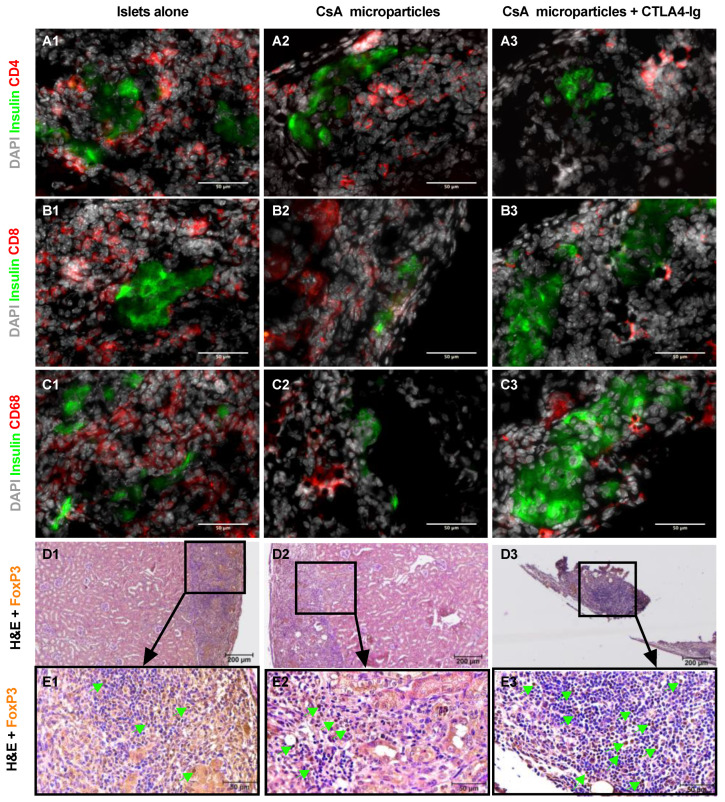
Immunohistochemistry of acute mouse islet allografts explanted 7 days post-transplant under the KC. Representative images of CD4^+^ (**A1**–**A3**), CD8^+^ (**B1**–**B3**) and CD68^+^ (**C1**–**C3**) cells. FoxP3 (brown, indicated by arrow) immunostaining was used to evaluate the presence of intra-islet graft FoxP3^+^ (Tregs) cells (**D1**–**D3**,**E1**–**E3**). Scale bars: 50 μm (**A**–**C**,**E**) and 200 μm (**D**).

To aid in elucidating the mechanisms by which CsA microparticles improve islet allograft survival, a series of acute allograft transplants was conducted and further analyzed using immunohistochemistry, proinflammatory cytokine secretion and inflammatory gene expression. Acute allogeneic islet grafts were stained for hematoxylin and eosin (H & E), insulin and glucagon, CD4, CD8, CD68, and FoxP3. We observed qualitatively higher mononuclear cellular infiltration into and surrounding the islets-alone allografts (Figure S2(A1)) compared to the CsA microparticles grafts (Figure S2(A2)) or in the combination of CsA microparticles + CTLA4-Ig grafts (Figure S2(A3)). Intact islets were observed in both CsA microparticles and CsA microparticles + CTLA4-Ig grafts (Figure S2(B2,B3)). CsA microparticles and CsA microparticles + CTLA4-Ig grafts displayed a significantly reduced presence of T cell populations such as CD4^+^ (Figure 4(A2,A3); Figure S2C) and CD8^+^ (Figure 4(B2,B3); Figure S2D) cells and macrophages (CD68^+^ cells) (Figure 4(C2,C3); Figure S2E) compared to the islet-alone grafts (Figure 4(A1,B1,C1); Figure S2C–E). Furthermore, intra-graft FoxP3-positive cells (T regulatory cells (Tregs)) were observed in the CsA microparticles (Figure 4(D2,E2)) and CsA microparticles + CTLA4-Ig treated grafts (Figure 4(D3,E3)). These results demonstrate that graft-localized CsA release reduces the infiltration of mononuclear cells.

**Figure 5:**
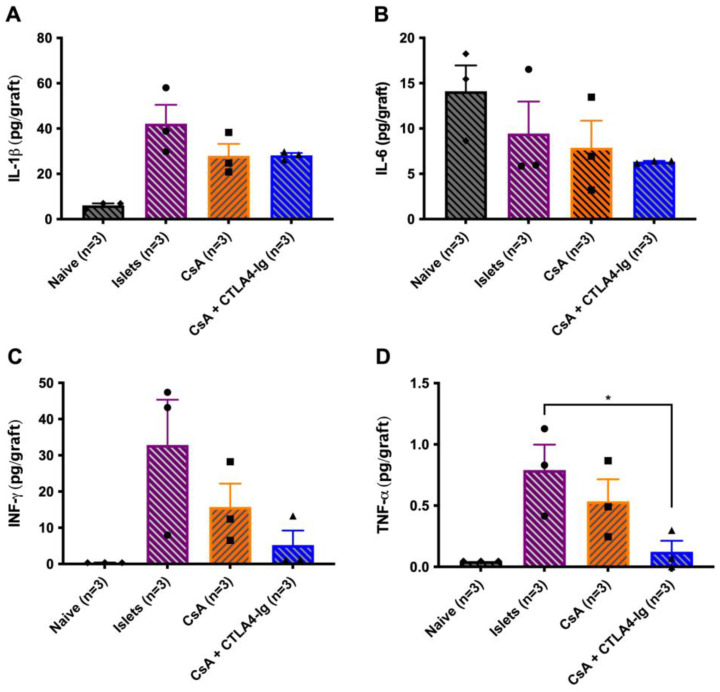
Intra islet graft proinflammatory cytokines analysis of acute allografts at 7 days post-transplant. Diabetic C57BL/6 mice were transplanted with BALB/c islets (500 islets) under the KC. Mouse recipient groups included were islets alone (*n* = 3, purple), CsA microparticles alone (*n* = 3, orange), and CsA microparticles + CTLA4-Ig (*n* = 3, blue). Kidney from nondiabetic, non-transplanted C57BL/6 mice (*n* = 3, black) served as a negative control. CTLA4-Ig (10 mg/kg) was administered i.p. at 0, 2, 4, and 6 days post-transplant. Graft-bearing kidneys were excised and homogenized to analyze the presence of IL-1β (**A**), IL-6 (**B**), INF-γ (**C**), and TNF-α (**D**). Data are expressed as mean ± SEM. * *p* < 0.05.

A cohort of acute allografts was analyzed for intra-graft proinflammatory cytokine profiles at 7 days posttransplant. IL-1β, IL-6, INF-γ, and TNF-α expression tended towards being reduced in both recipients treated by CsA microparticles and those treated by CsA microparticles + CTLA4-Ig compared to islet-alone recipients (Figure 5A–D). Of note, CsA microparticles + CTLA4-Ig grafts had significantly lower expression of TNF-α than islets alone grafts (*p* < 0.05). IL-10, IL-12p70, and KCGRO expression was also measured; however, they were undetected in all grafts.

**Figure 6:**
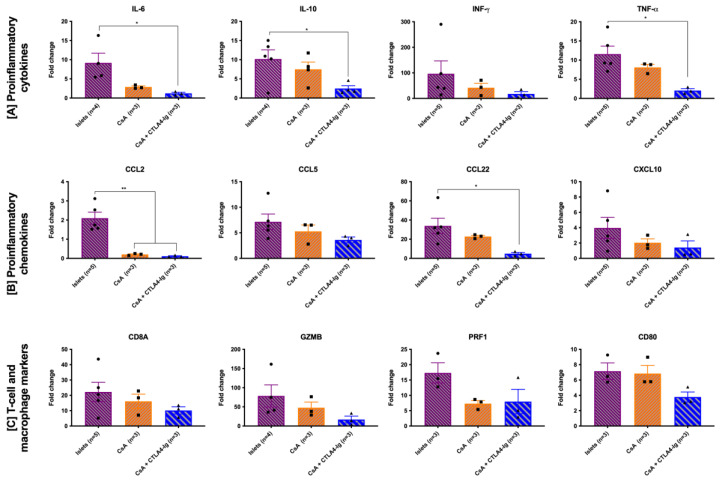
Gene expression from acute 7-day intra-islet grafts. Diabetic C57BL/6 mice were transplanted with BALB/c islets (500 islets) under the KC. Mouse recipient groups included were islets alone (purple), CsA microparticles alone (orange), and CsA microparticles + CTLA4-Ig (blue). Kidneys from sham-transplanted C57BL/6 mice (*n* = 3) served as a negative control. CTLA4-Ig (10 mg/kg) was administered i.p. at 0, 2, 4, and 6 days post-transplant. Fold change in the expression of proinflammatory cytokines *IL-6*, *IL-10*, *INF-γ*, and *TNF-α* (**A**); proinflammatory chemokines *CCL2*, *CCL5*, *CCL22*, and *CXCL10* (**B**); and T-cell and macrophage markers *CD8A*, *GZMB*, *PRF1*, and *CD80* (**C**). Data are expressed as mean ± SEM. * *p* < 0.05; ** *p* < 0.01.

A final cohort of recipients was analyzed for the intra-graft mRNA expression of proinflammatory cytokines, chemokines, and T-cell and macrophage markers using real-time RT-PCR at 7 days posttransplant. The reduced mRNA expression of proinflammatory cytokines (*IL-6*, *IL-10*, *INF-γ* and *TNF-α*) and proinflammatory chemokines (*CCL2*, *CCL5*, *CCL22*, and *CXCL10*) was observed in CsA-microparticle-treated recipients compared to the islet-alone recipients (Figure 6A,B). Most notably, *IL-6*, *IL-10*, *TNF-α*, *CCL2*, and *CCL22* mRNA expression were significantly reduced in the recipients treated with CsA microparticles + CTLA4-Ig compared to the islet-alone recipients (Figure 6A,B, *p* < 0.05, *p* < 0.01). Furthermore, T cell markers (*CD8A*, *GZMB*, and *PRF1*) and macrophage marker (*CD80*) mRNA expression levels were modestly decreased in the recipients treated with CsA microparticles and those treated with CsA microparticles + CTLA4-Ig compared to the islet-alone recipients (Figure 6C).

### 3.5. Histological Characterization of Long-Term Islet Allografts

**Figure 7:**
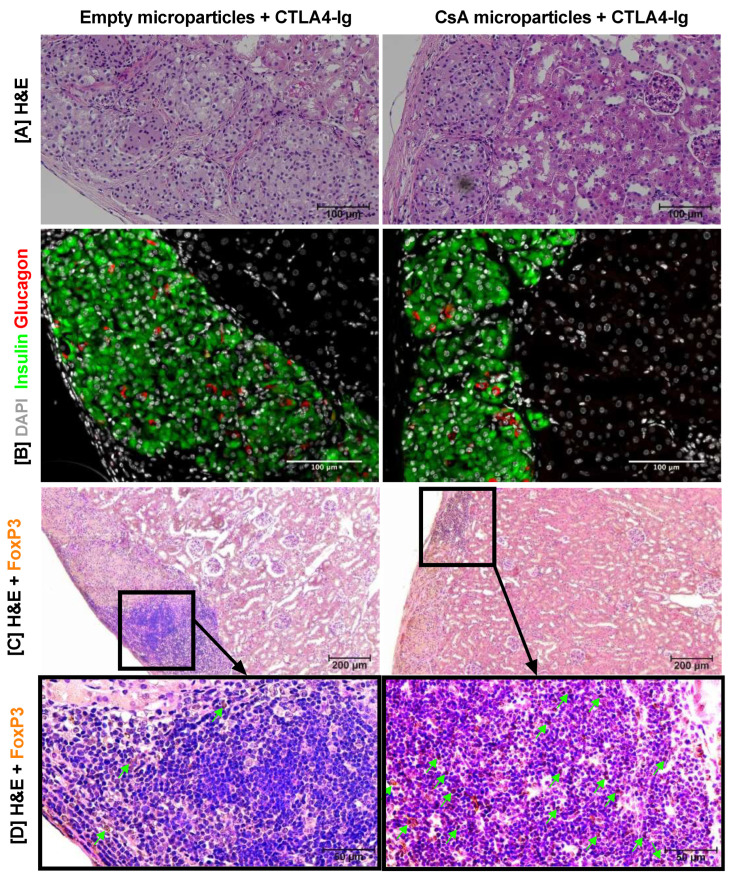
Immunohistochemistry of long-term islet allografts explanted at 214 days post-transplant under the KC. Representative image of islets using H & E staining (**A**). Representative immunofluorescence of islets in both CsA microparticles + CTLA4-Ig and empty microparticles + CTLA4-Ig grafts. Insulin is represented as green and glucagon as red (**B**). Representative FoxP3 immunostaining plus H & E staining of the area surrounding the islets (**C**); FoxP3 immunostaining (brown, indicated by arrow) in both CsA microparticles + CTLA4-Ig and empty microparticles + CTLA4-Ig grafts (**D**). Scale bars: 50 μm (**D**), 100 μm (**A**,**B**), and 200 μm (**C**).

The H & E staining of long-term grafts (214 days) from both CsA microparticles + CTLA4-Ig and empty microparticles + CTLA4-Ig demonstrated the presence of intact islets (Figure 7A). Furthermore, immunohistochemical staining demonstrated that the grafts predominantly consisted of insulin-positive β-cells with a few glucagon-positive α-cells (Figure 7B). Additionally, intragraft FoxP3^+^ cells (Tregs) were observed in grafts treated with both empty microparticles + CTLA4-Ig and CsA microparticles + CTLA4-Ig grafts (Figure 7C,D); however, substantially more FoxP3^+^ cells were observed in the grafts collected from animals treated with CsA microparticles + CTLA4-Ig.

### 3.6. CsA Microparticles + CTLA4-Ig Treatment Generated Operational Tolerance

**Figure 8:**
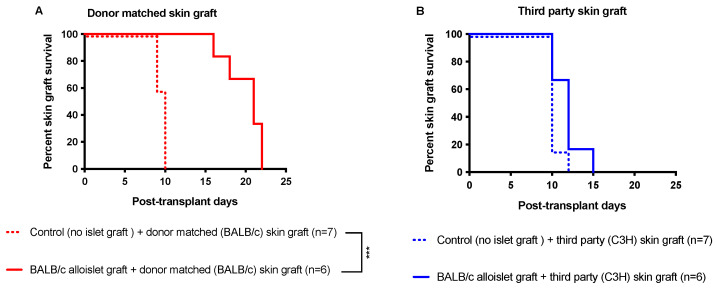
Skin graft survival. Skin graft transplants were conducted on recipients treated with islets + CsA + CTLA4-Ig at 100 days post-transplant to investigate the transplant tolerance. Diabetic C57BL/6 mice were transplanted with BALB/c islets (500 islets) under the KC. CTLA4-Ig (10 mg/kg) was administered i.p. at 0, 2, 4, and 6 days post-transplant. Survival of skin grafts was analyzed on islets + CsA microparticles + CTLA4-Ig recipients, and control recipients (non-islet-transplanted recipients and those treated with no CsA + CTLA4-Ig) (**A**,**B**). *** *p* < 0.001.

To confirm that CsA microparticles + CTLA4-Ig treatment generated operational tolerance, we performed skin graft transplants on islet allograft recipients at 100 days posttransplant. We observed that BALB/c skin graft (donor-matched) rejection was significantly delayed in recipients treated with islet + CsA microparticles + CTLA4-Ig (average skin graft rejection was 20.0 ± 1.0 days) compared to the control recipients (non-islet transplanted and no CsA + CTLA4-Ig treated recipients, average skin graft rejection 9.57 ± 0.20 days) (Figure 8A, *p* < 0.001, log-rank). In contrast, C3H skin (third-party skin) graft rejection was comparable between recipients treated with islet + CsA microparticles + CTLA4-Ig (average skin graft rejection 11.83 ± 0.74 days) and control recipients (recipients that were non-islet transplanted and those treated with no CsA + CTLA4-Ig, average skin graft rejection 10.29 ± 0.28 days) (Figure 8B). Syngeneic skin grafts were accepted (Figure S3(A1)). BALB/c skin grafts (donor-matched skin) (Figure S3(B1)) were rejected gradually, whereas C3H skin grafts (third-party skin) (Figure S3(C1)) were rapidly rejected in recipients treated with islets + CsA microparticles + CTLA4-Ig. The H & E staining of rejected BALB/c skin (Figure S2(B2)) and C3H skin (Figure S3(C2)) showed the absence of an intact epithelial layer compared to the naïve control skin (Figure S3(B3–C3)), indicating the complete rejection of the skin grafts. Interestingly, alloislet-transplanted recipients maintained normoglycemia until BALB/c skin graft rejection. The rejection of BALB/c skin appears to have triggered the rejection of the islet graft (Figure S4). The rapid rejection of third-party skin grafts together with delayed donor skin graft rejection indicated that graft-localized CsA-eluting microparticles + CTLA4-Ig promoted operational tolerance.

## 4. Discussion

Allogeneic islet transplantation has successfully restored physiological glucose homeostasis in selected patients with T1D [24]. However, multiple factors, such as oxidative stress, inflammatory insults, immune cells attack and systemic immunosuppression toxicity, cause damage to the transplanted islets, and patients often need to return to exogenous insulin administration [25]. In addition, the recurrence of islet autoimmunity may further accelerate the destruction of transplanted β-cells [26]. Anti-inflammatory and immunosuppressive medications like etanercept, ATG, rapamycin, tacrolimus, and CsA are clinically used to prevent alloislet rejection. Nevertheless, these drugs are associated with undesirable side effects on the transplanted cells and the patient’s health [27,28]. The induction of local immunotolerance is an attractive strategy to prevent islet allograft rejection, which may improve graft function and broaden cellular therapy’s applicability while minimizing adverse side-effects of systemic immunosuppression [18,29,30]. Several strategies have been established to evoke immunoprotection by co-delivering anti-rejection drugs with particles/scaffolds to the islet grafts [3,18,31,32,33]. Immunomodulating drug-loaded microparticles provide multifaceted tools to locally modulate immune responses and represent an exciting tactic to aid cell transplantation applications. Furthermore, it is possible to load multiple drugs into a single carrier to target various pathways of the immune system simultaneously and adjust drug release kinetics by modifying the co-polymer ratios. The local release of therapeutic drugs is favored over systemic administration as a means to offset the harmful or adverse immune-dampening effects when left to circulate systemically [17,26]. Therefore, we developed and characterized the impact of islet-graft-localized CsA-eluting PLGA microparticles on modulating the inflammatory responses in the islet transplant milieu.

CsA is a calcineurin inhibitor and its potent immunosuppressive properties have profoundly improved solid organ transplant outcomes [34]. CsA exerts immunomodulatory effects by blocking interleukin (IL)-2–dependent proliferation and the differentiation of T cells, and, moreover, cyclosporine A–cyclophilin D complex formation stabilizes mitochondrial function and prevents cell death [6,7]. However, the diabetogenic effect and nephrotoxicity remain restricting the widespread use of CsA [14,20]. Conversely, a high dose of CsA (50 mg/kg) is associated with morbidity and mortality in animal models [14], whereas the therapeutic (5 mg/kg) [6] or subtherapeutic (1 mg/kg) [15] systemic doses of CsA are insufficient to prevent islet allograft rejection. Furthermore, CsA exhibits low oral bioavailability owing to its poor biopharmaceutical properties, such as its low aqueous solubility and low permeability, which pose challenges in making a safe and effective delivery system [35]. Consequently, there is a need for the development of a novel formulation strategy to encapsulate CsA with better bioavailability and fewer side effects. To the best of our knowledge, our study is the first to show the effect of CsA-encapsulated PLGA particles in the context of islet transplantation. Drug encapsulation efficiency is affected by many factors, including processing conditions, surfactants, solvents, molecular weight, surface functionalization, loading concentrations, and the nature of the drug and its interaction with the PLGA [36]. We previously observed poor Dex encapsulation efficiency in PLGA microparticles (14.6 ± 1.7%) due to poor interactions between the Dex and PLGA [18]. However, in this current study, we achieved improved CsA encapsulation efficiency of 89.02 ± 1.46% in PLGA microparticles by modifying our emulsion process. We used a pre-cooled PVA solution to make the emulsion; this pre-cooling of PVA prevents solvent evaporation during the emulsion process. Unlike Dex, CsA is a cyclic peptide that has free functional groups that have a higher affinity interaction with the PLGA, which validates earlier studies demonstrating that the interaction of the drug and polymer has a pivotal role in achieving a better drug encapsulation [36,37].

Our data confirmed that the administration of a single dose of 4 mg of CsA microparticles to the localized graft site is safe, did not comprise islet graft function, and yielded allograft protection for 5 more weeks, than the allografts containing empty microparticles (no CsA). While the effective systemic dose of CsA is 5 mg/kg, 1 mg/kg is considered a subtherapeutic dose. However, in this study, we have shown that the local release of 0.04 to 0.1 mg/kg of CsA, which is 50 to 1250 times lower than the therapeutic systemic dose, has been able to induce allograft protection without inducing cytotoxicity or affecting islet functionality. However, we did not observe a durable graft function with this monotherapy, mirroring the observations of Arita et al., who showed that neither pravastatin- nor CsA-alone-treated recipients prolonged alloislet graft survival [14].

Since higher doses of CsA are associated with impaired glucose tolerance, decreased insulin content, and a decreased β-cell volume [10], we speculated that combining localized CsA microparticles with other immunomodulatory delivery modalities (i.e., CTLA4-Ig) would potentiate allograft protection. CTLA4-Ig effectively attenuates T-cell activation by competing with the costimulatory molecule CD28 to bind with ligands CD80 and CD86 on antigen-presenting cells (APCs), for which it has a higher affinity and avidity than CD28 [38]. Indeed, previous islet allograft studies demonstrated that CTLA4-Ig costimulatory blockade alone yielded prolonged graft survival in approximately one-third of the murine recipients [39,40]. In a large-animal non-human primate study, CTLA4-Ig therapy was demonstrated to prolong islet allograft survival in 40% of the recipients while demonstrating CTLA4-Ig’s effectiveness in suppressing both humoral and cellular immune responses [41]. Islet allograft survival and euglycemic percent rate can be augmented when CTLA4-Ig is combined with other immunosuppressive agents such as Basiliximab (anti-IL-2R mAb), Sirolimus, and 3A8 (anti-CD40 mAb) [42].

Recently, our group demonstrated that the co-localization of Dex microparticles + islet graft resulted in prolonged allograft function when combined with the low dose (10 mg/kg) administration of CTLA4-Ig i.p. [18]. Therefore, we examined the ability of CTLA4-Ig to work in concert with CsA microparticle co-delivery to improve allograft survival. A total of 54% of the recipients of allogeneic islets + CsA microparticles co-localization and short-term treatment of CTLA4-Ig maintained normoglycemia up to 214 days. In contrast, only 25% of the recipients of empty microparticles + CTLA4-Ig maintained normoglycemia for up to 214 days. Recipients treated with CsA microparticles plus CTLA4-Ig had double the long-term euglycemia prevalence compared to recipients of empty microparticles + CTLA4-Ig monotherapy.

We hypothesized that localized CsA delivery to the islet grafts site increases primarily ß-cell survival by favorably modulating the transplant of inflammatory milieu. It is well known that inflammatory mediators such as proinflammatory cytokines, proinflammatory chemokines, and complement activation products contribute to the early loss of islets and negatively impact islet graft functions [43]. We observed that T-cell (CD4^+^ and CD8^+^ cells) and macrophage (CD68^+^ cells) populations were significantly reduced in those treated with CsA microparticles or CsA microparticles + CTLA4-Ig compared to the islet-alone recipients. Previous studies demonstrated that the migration of activated CD8^+^ T cells to the islet grafts site triggers more damage and eventually destroys the transplanted allogeneic islet by releasing granzyme B (GZMB) and perforins (PRF1) [44]. CD4^+^ T cells indirectly contribute to graft rejection by boosting CD8^+^ T cells and secreting more inflammatory cytokines, such as interferon-gamma (IFN-γ) and tumor necrosis factor-alpha (TNF-α) [29]. Our study also concurs with earlier observations that those treated with CsA microparticles + CTLA4-Ig showed a marked reduction in the mRNA expression of intra-islet graft cytokines (*IL-1ß*, *IL-6*, *IL-10*, *INF-γ*, and *TNF-α*) and proinflammatory chemokines (*CCL2*, *CCL5*, *CCL22*, and *CXCL10*), known inducers of ß-cell apoptosis [45,46]. Similarly, proinflammatory chemokines (*CXCL10*, *CCL2*, *CCL5*, and *CCL22*) elicit poor islet allograft function [47,48]. These chemokines play an essential role in T1D disease progression and the islet graft rejection [30]. We also observed the reduced expression of *CD8A*, *GZMB*, *PRF1*, and *CD80* in those treated with CsA microparticles + CTLA4-Ig. Thus, these observations prove that our CsA microparticles + CTLA4-Ig approach plays a vital role in altering the localized immune response while yielding improved allograft survival.

We observed long-term islet allograft function in those treated with CsA microparticles + CTLA4-Ig. To determine whether operational tolerance was induced in these recipients, we performed skin grafts and compared them to control recipients (non-transplanted and non-CsA-treated). We observed that BALB/c skin graft (donor-matched skin) rejection was significantly delayed in recipients of islets + CsA microparticles + CTLA4-Ig compared to the control recipients. However, C3H skin (third-party skin) rejection was comparable between recipients of islets + CsA microparticles + CTLA4-Ig treatment and control recipients. Our skin graft results are consistent with previous reports whereby naïve B6 mice rejected BALB/c skin allografts at 14 days posttransplant, whereas intrahepatic islet-transplanted tolerant B6 mice showed a modest prolongation of skin allograft survival for up to 17 days posttransplant [22]. Our observations align with those of others who reported that tolerance to Fas ligand chimeric with streptavidin (SA-FasL)-engineered islet grafts is donor-specific and systemic at the induction phase. Similar to our data, tolerance was antigen- and tissue-specific, as SA-FasL-engineered islets failed to protect third-party islet, donor-matched, and third-party skin recipients [23]. The rejection of donor-matched skin grafts, but not the third-party skin grafts, also culminated in the rejection of SA-FasL-engineered islet grafts. Similarly, in our study, we observed that alloislet recipients maintained normoglycemia until the BALB/c skin rejection, indicating that BALB/c skin graft rejection elicits vigorous allogeneic immune responses that simultaneously reject islet allografts [23]. Taken together, our skin graft transplant findings suggest that our CsA microparticles + CTLA4-Ig treatments generated an operational tolerance.

We speculated that Tregs expression at the graft site could be causal for the observed transplant tolerance. It has been reported that Treg cells traffic to allogeneic pancreatic islets immediately post-transplantation in response to inflammatory cues, where they manifest their immunoregulatory function within the graft microenvironment [23]. Moreover, Tregs play a crucial role in maintaining immune homeostasis and peripheral tolerance to foreign antigens in humans [49,50]. Also, it has been reported that Tregs in the pancreas inhibit the actions of autoreactive T cells, thereby preventing diabetes progression [51]. Our recent study also demonstrated that a Dex-eluting microparticles + CTLA4-Ig islet allograft contained more FoxP3^+^ Tregs. Indeed, in this present study, we observed the abundant presence of intragraft FoxP3^+^ Tregs in both acute and long-term CsA microparticles + CTLA4-Ig islet allografts.

Our results illustrate that the administration of intragraft CsA microparticles in combination with CTLA4-Ig treatment promotes graft survival. However, to gain a better understanding of the mechanism(s) by which CsA microparticles and CTLA4-Ig treatment promote immunomodulation in the graft area, it will be essential to characterize and quantify the immune cell phenotypes within the graft area. For future studies, our focus will be on characterizing and quantifying immune cell phenotypes at different time points, with a particular emphasis on FoxP3^+^ Tregs. Of note, recent research has demonstrated that murine CD4^+^ FoxP3^+^ T cells can be classified into two subpopulations, with distinct FoxP3 localization based on the expression of CD25; CD4^+^ CD25^+^ T cells expressing FoxP3 in the nucleus, whereas CD4^+^ CD25^−^ T cells express FoxP3 in the cytoplasm [52,53]. In the present study, we observed both the nuclear and cytoplasmic localization of FoxP3 within long-term functional allografts, consistent with the findings of other modalities to prevent murine islet allograft rejection [54,55]. As such, we will also characterize and quantify CD4^+^ CD25^+^ Foxp3^+^ Treg and CD4^+^ CD25^−^ Foxp3^+^ Treg populations to better understand the role of these populations in murine islet allograft engraftment and rejection. Additionally, we will study the expression of an array of proinflammatory and immunomodulatory genes using single-cell RNA sequencing at various time intervals post-transplant to help us determine when the CsA microparticle treatment alters the proinflammatory milieu and how it enhances allograft function. In addition, gene and protein analysis will allow us to identify the mechanism(s) involved in rejecting or inducing immune tolerance. This understanding will aid in paving the way for establishing the application of localized drug delivery in the clinical settings. Our ultimate goal is to reduce the requirement of systemic immunosuppression in transplant settings to avoid side effects and improve the quality of life of patients. For this reason, we aimed to provide a reduced dose of CsA (below the subtherapeutic dose of CsA, 0.1 mg/kg) locally to the graft site to improve the allograft function, and effectively demonstrated that a localized average daily delivery of 0.1 mg/kg of CsA (below the subtherapeutic dose) provides alloislet protection for 5 weeks. Furthermore, the combination of localized CsA delivery plus short-term CTLA4-Ig treatment resulted in improved islet allograft outcomes (maintained euglycemia for 214 days, study end point). We believe systemic administration of CsA at such a low dosage (0.1 mg/kg) would not be efficacious and hence, we believed it was not ethical to include another group of control mice based on the previous report where it was shown that therapeutic (5 mg/kg) [6] or subtherapeutic (1 mg/kg) [15] systemic doses of CsA are insufficient to provide allograft protection.

Despite these limitations, our findings suggest that localized CsA drug delivery via microparticle elution provides a feasible therapeutic platform to deliver immunomodulatory drugs in a controlled and favorable manner at the islet graft site. This therapy is a safe and effective strategy for minimizing systemic immunosuppression and promotes durable islet allograft function with temporary systemic immunosuppressive therapy. Furthermore, treatment with CsA plus CTLA4-Ig generated operational tolerance. We anticipate that this approach may be suitable for developing long-lasting immunomodulatory agents that effectively boost islet allograft survival without the need for systemic immunosuppression.

## Data Availability

All datasets and analyses contained within this study can be obtained from the corresponding author upon reasonable request.

## Supplementary Materials

The following supporting information can be downloaded at: https://www.mdpi.com/article/10.3390/pharmaceutics15092201/s1. Figure S1. In vivo release of CsA from CsA microparticles combined with collagen type I implanted under the KC over 21 days (A–C). Figure S2. Immunohistochemistry of acute mouse islet allografts explanted 7 days post-transplant under the KC. Figure S3. Representative macroscopic images of skin grafts on mice treated with allogeneic BALB/c islets + CsA microparticles + CTLA4-Ig. Syngeneic skin graft (C57BL/6 mouse skin to C57BL/6 mouse) was accepted in recipient mice [A1], and also, observed an intact re-epithelialization in the accepted skin [H&E, A2], Figure S4. Blood glucose levels of recipient mice treated with allogeneic BALB/c islets + CsA microparticles + CTLA4-Ig post-skin grafting (colored lines indicate the individual mouse blood glucose). Table S1. List of FAM-MGB (FAM: Fluorescein amidites, MGB: Minor groove binder) primer-probe mixes (Applied Biosystems-Thermo Fisher Scientific, MA, USA) was used in Real-time RT-PCR.
