# Supplementary material for: Long-Term Survival and Induction of Operational Tolerance to Murine Islet Allografts by Co-Transplanting Cyclosporine A Microparticles and CTLA4-Ig
Source: Pharmaceutics. 2023 Aug 25;15(9):2201. doi: 10.3390/pharmaceutics15092201 (PMC10537425; doi:10.3390/pharmaceutics15092201)
Supplement: Supplementary file 1 [file pharmaceutics-15-02201-s001.zip › pharmaceutics-2570336-supplementary.pdf]

pharmaceutics

MDPI

Supplementary Materials: Long-Term Survival and Induction of Operational Tolerance to Murine Islet Allografts by Co-Transplanting Cyclosporine A Microparticles and CTLA4-Ig

Purushothaman Kuppan, Jordan Wong, Sandra Kelly, Jiaxin Lin, Jessica Worton, Chelsea Castro, Joy Paramor, Karen Seeberger, Nerea Cuesta-Gomez, Colin C. Anderson, Gregory S. Korbutt and Andrew R. Pepper

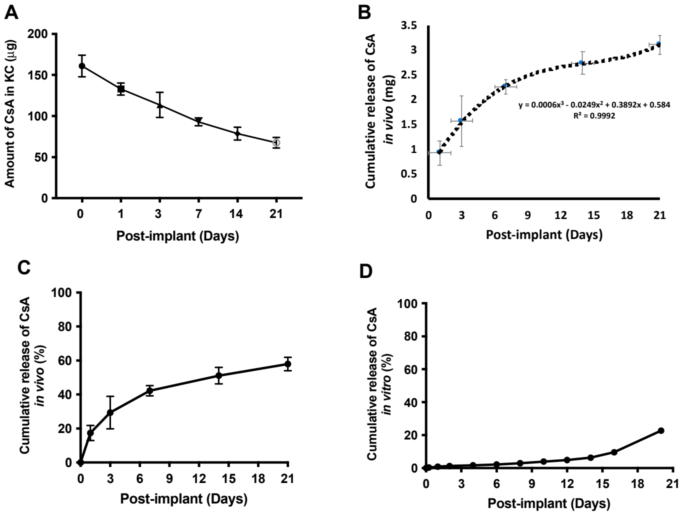

Figure S1. *In vivo* release of CsA from CsA microparticles combined with collagen type I implanted under the KC over 21 days (A—C). *In vitro* release of CsA from CsA microparticles in PBS over 21 days (D).

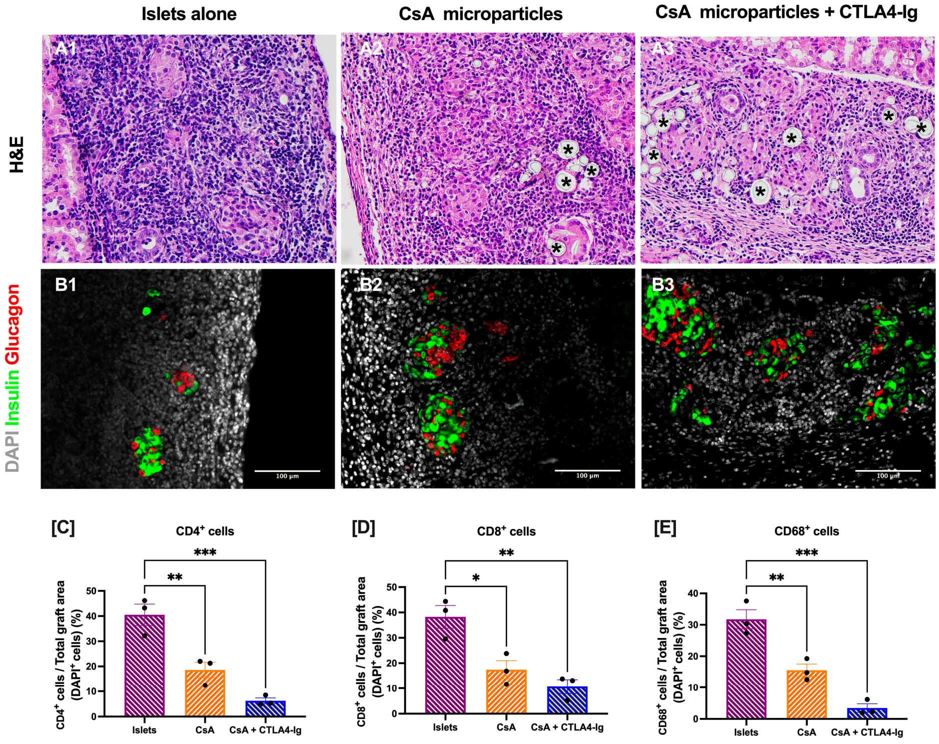

Figure S2. Immunohistochemistry of acute mouse islet allografts explanted 7 days post-transplant under the KC. Representative H&E staining of acute allogenic grafts revealed the presence of intact islet clusters with minimal infiltration of mononuclear cells observed in CsA microparticles [A2] and CsA microparticles + CTLA4-Ig [A3] grafts, compared to islets alone grafts [A1]. Immunostaining of allogeneic grafts demonstrated that the intact islet clusters were positive for insulin (green) and glucagon (red) [B1—B3]. CsA microparticles and CsA microparticles + CTLA4-Ig grafts displayed significantly a reduced presence of T cell populations such as $CD4^+$ [C] and $CD8^+$ [D] cells and macrophages ($CD68^+$ cells) [E] compared to the islet alone grafts [C-E] ($p < 0.05$, $p < 0.01$, $p < 0.001$, Anova). *p<0.05, **p<0.01, ***p<0.001

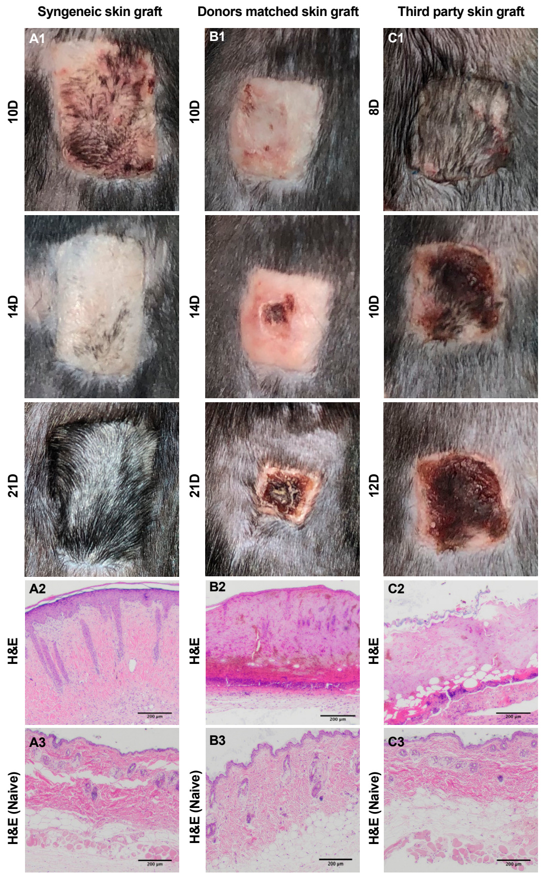

Figure S3. Representative macroscopic images of skin grafts on mice treated with allogeneic BALB/c islets + CsA microparticles + CTLA4-Ig. Syngeneic skin graft (C57BL/6 mouse skin to C57BL/6 mouse) was accepted in recipient mice [A1], and also, observed an intact re-epithelialization in the accepted skin [H&E, A2]. Delayed BALB/c skin graft (donors matched skin) [B1] rejection was observed in recipients treated with islets + CsA microparticles + CTLA4-Ig compared to the C3H skin graft (Third-party skin) [C1] rejection in the same recipients. H&E staining of rejected BALB/c skin graft [B2] and C3H skin graft [C2] demonstrating the absence of an intact epithelial layer compared to the naïve control skin grafts [B3 and C3]. Scale bars; 200 μm.

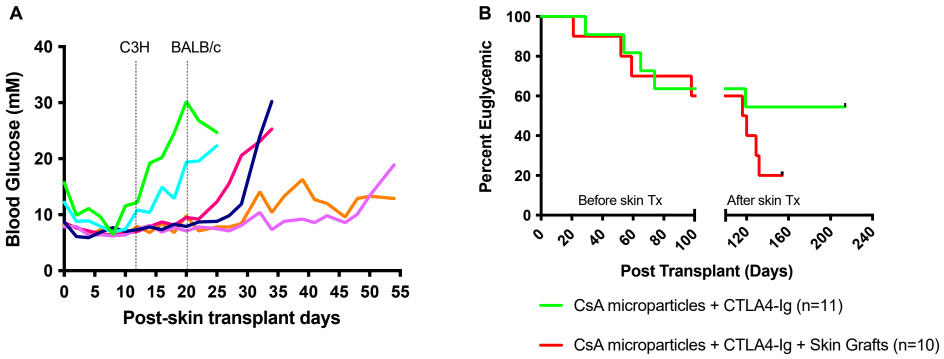

Figure S4. Blood glucose levels of recipient mice treated with allogeneic BALB/c islets + CsA microparticles + CTLA4-Ig post-skin grafting (colored lines indicate the individual mouse blood glucose). Black dotted lines demonstrate an average day of C3H (11.83 ± 0.74 day) and BALB/c (20.0±1.0 day) skin graft rejection. Islet allograft survival, percentage euglycemia in mouse recipients transplanted with CsA microparticles + CTLA4-Ig before and after skin grafting [B].

Table S1. List of FAM-MGB (FAM: Fluorescein amidites, MGB: Minor groove binder) primer-probe mixes (Applied Biosystems-Thermo Fisher Scientific, MA, USA) was used in Real-time RT-PCR.

Gene Symbol	Assay ID
IL-6	Mm00446190_mL
IL-10	Mm01288386_mL
INF-γ	Mm01168134_mL
TNF-α	Mm00443258_mL
CCL2	Mm00441242_mL
CCL5	Mm01302428_mL
CCL22	Mm00436439_mL
CXCL10	Mm00445235_mL
CD8A	Mm01182107_gL
GZMB	Mm00442837_mL
PRF1	Mm00812512_mL
CD80	Mm00711660_mL

1. Preparation and characterization of CSA eluting PLGA microparticles

Cyclosporine A (CsA, Mw 1202.6 g/mol, AdooQ Bioscience, CA, USA) eluting poly(lactic-co-glycolic acid) (PLGA: Mw 30,000—]60,000 g/mol; 50:50 ratio, Sigma-Aldrich, ON, Canada) microparticles were prepared by a modified single emulsion (O/W, oil-in-water emulsion) solvent evaporation technique as previously described [1–3]. Briefly, PLGA (5% weight/volume) and CsA (5 mg/mL) were dissolved in dichloromethane (DCM, Sigma-Aldrich, ON, Canada). Subsequently, polymer-drug solution was emulsified using 10 mL of pre-cooled 4% polyvinyl alcohol (PVA, 80% hydrolyzed, Mw 9000-10000, Sigma-Aldrich, ON, Canada) solution with vigorous stirring for 5 min in a magnetic stirrer (VWR International, ON, Canada). Finally, all the contents were transferred into a 200 mL of 2% PVA solution and continuously stirred (high torque stirrer, Caframo®, BDC6015, ON, Canada) at 1000 rpm for an hour at room temperature. This process aids in completely evaporating the DCM while hardening the microparticle particles. Microparticles were collected by centrifugation (Allegra® X-15R centrifuge, Beckman coulter, NH, USA) at 3000 rpm for 5 min at 4 °C followed by lyophilized (Dura-Dry™ MP, FTS Systems, NY, USA). Drug-free microparticles (empty microparticles) were prepared in the same manner as described above, however, CsA was not included in the solvent. Empty PLGA microparticles were used in transplant studies to demonstrate their safety, tolerability and inability alone to confer protection from alloimmune responses.

CsA-loaded PLGA microparticle morphology was characterized by scanning electron microscopy (ZEISS EVO 10 Scanning Electron Microscope, Zeiss, NY, USA). Lyophilized CsA microparticles were spread onto carbon tape, coated with gold sputtering (Hummer 6.2 sputter coater, LADD Research Industries, VT, USA) and imaged. The particle diameter was measured manually using SEM images and a histogram was plotted. The amount of CsA entrapped within the microparticles (encapsulation efficiency) was quantified by high-performance liquid chromatography (HPLC) (Agilent Technologies, 1200 series, CA, USA) [4]. Briefly, 10 mg of lyophilized CsA particles (n = 15) were dissolved in acetonitrile (Sigma-Aldrich, ON, Canada): methanol (Fisher Scientific, ON, Canada) mixture (8:2) to release the CsA followed by centrifuged (Allegra® X-15R centrifuge, Beckman coulter, NH, USA) at 3000 rpm for 5 min. From this, a clear 10 µL solution was injected into the C 18 column, eluted at 0.25 mL/minute using acetonitrile: water (65:35) that detects the unknown CsA concentrations at 210 nm [4]. The column temperature was maintained at 60 °C. A series of known CsA concentrations between 10 µg/mL–1000 µg/mL were run parallel to extrapolate the unknown sample concentrations.

In vitro drug release was conducted to understand the release pattern and kinetics of CsA microparticles [2,3,5,6]. In brief, 30 mg of lyophilized CsA microparticles (n = 4) were dispersed in 1 mL of phosphate-buffered saline (PBS, Thermo Fisher Scientific, ON, Canada) and maintained at 37 °C. Two hundred microliters of samples were collected and replaced with the same amount of fresh PBS for 30 days. Samples were analyzed at 210 nm using a Multiskan SkyHigh microplate spectrophotometer (Thermo Fisher Scientific, ON, Canada) for the presence of CsA. A series of known CsA concentrations between 10 µg/mL–100 µg/mL were run parallel to extrapolate the unknown sample concentrations.

2. Islet isolation, transplantation and metabolic follow-up

All animal studies were in accordance to the Canadian Council of Animal Care and guidelines obtained from the institutional ethical committee of the University of Alberta. All mice were maintained in a clean, sterile and pathogen-free environment and had access to *ad libitum* of water and pelleted food. All donors were male BALB/c mice (The Jackson Laboratory, Bar Harbor, ME, USA) between 8 and 12 weeks and weighed between 22 and 27 g. Mouse islets isolation and purification were conducted based on the previously described methodology [7]. Briefly, collagenase (5 mg/mL) and thermolysin (0.2 mg/mL) (Liberase™ TL Research Grade, Roche Diagnostics, Mannheim, Germany) in Hank's Balanced Salt Solution (HBSS, Sigma-Aldrich, ON, Canada) was injected through the common bile duct to distend the pancreas in situ while blocking the duct entering at the duodenum. The distended pancreata were subsequently digested at 37 °C for 14 min in a shaking water bath (Thermo Fisher Scientific, ON, Canada) at 50 rpm. Islets were purified from the pancreatic digest through histopaque density gradient (1.108, 1.083, and 1.069 g/mL, Sigma-Aldrich, ON, Canada) centrifugation at 3000 rpm for 12 min. Collected islets were washed with HBSS and cultured in Connaught Medical Research Laboratories (CMRL-1066, Mediatech, Manasses, VA) supplemented with fetal bovine serum (10%), L-glutamine (100 mg/L), penicillin (112 kU/L), streptomycin (112 mg/L), and HEPES (25 mmol/L) at pH 7.4 for an hour prior to transplantation.

Prior to transplant, recipient male BALB/c and C57BL/6 mice (The Jackson Laboratory, Bar Harbor, ME, USA), between 8—12 weeks of age, were injected with 180 mg/Kg streptozotocin (STZ, Sigma-Aldrich, ON, Canada) reconstituted in acetate buffer (pH 4.5), intraperitoneally (i.p) to induce diabetes [3,8]. Animals with blood glucose readings >18 mmol/L, measured using OneTouch UltraMini glucose meter (LifeScan, Burnaby, BC, Canada), for two consecutive days were considered diabetic and utilized for subsequent transplant studies. Syngeneic islet transplantation studies were conducted to assess the adverse effect of CsA microparticles. Diabetic BALB/c mice were transplanted with either 4 mg (~ 356 µg of CsA) of CsA microparticles + 500 BALB/c islets (n =3) or islets alone (n =3) under the KC [7]. A separate cohort of allogeneic mouse islets transplants was conducted by co-delivering 500 BALB/c islets with 4 mg CsA microparticles under the KC of

diabetic C57BL/6 mice. Allogeneic experimental groups were (1) empty microparticles (no CsA) + islets (*n* = 8), (2) CsA microparticles + islets (*n* = 7), (3) empty microparticles + islets + CTLA4-Ig (*n* = 8), and (4) CsA microparticles + islets + CTLA4-Ig (*n* = 11). CTLA4-Ig (10 mg/kg, Biocell, West Lebanon, NH) was administered i.p on days 0, 2, 4, and 6 posttransplant. After transplantation, the recipient's blood glucose was monitored three times per week. Allograft rejection was defined as two consecutive blood glucose readings greater than 18.0 mM. In both syngeneic and allogeneic studies mice that maintained normoglycemia at 35 days and 100 days, posttransplant respectively were subjected to an intraperitoneal glucose tolerance test (IPGTT). Naïve, nondiabetic C57BL/6 (*n* = 7) and BALB/c (*n* = 3) mice served as controls. Overnight fasted mice were injected 3 g/kg of glucose (DMVet, Coaticook, QC, Canada) i.p. and blood glucose was measured at 0, 15, 30, 60, 90 and 120 min. Glucose clearance was expressed and compared by blood glucose area under the curve (AUC) values. Subsequently, allograft recipients that maintained euglycemia up to 214 days post-transplant were electively subjected to survival nephrectomize to confirm graft-dependent function. For histological assessment, graft-bearing kidneys were immediately fixed in 10% formalin (BDH Laboratory Supplies, VWR International, AB, Canada). Once recipients reverted to a pre-transplant hyperglycemic level, confirmed by two consecutive days, animals were euthanized.

3. Intra-islet graft proinflammatory cytokine analysis and gene expression analysis

Acute allotransplant studies were conducted where diabetic C57BL/6 mice received 500 BALB/c islets + 4 mg CsA microparticles (*n* = 3) or 500 BALB/c islets + 4 mg CsA microparticles + CTLA4-Ig (10 mg/kg, 0, 2, 4, and 6 posttransplant). Diabetic mice that received islets alone served as a positive control (*n* = 3) and non-transplanted diabetic C57BL/6 mice (*n* = 3) served as a negative control. After seven days, graft-containing kidneys were removed and analyzed for intraislets graft proinflammatory cytokines via Mouse Pro-inflammatory 7-Plex Ultra-Sensitive Kit (Meso Scale Diagnostics, Rockville, MD) [3,7].

Acute allotransplant studies were conducted where diabetic C57BL/6 mice received 500 BALB/c islets alone (*n* = 3), or 500 BALB/c islets + 4 mg CsA microparticles (*n* = 3) or 500 BALB/c islets + 4 mg CsA microparticles + CTLA4-Ig (10 mg/kg, 0, 2, 4, and 6 posttransplant). Naïve, sham transplanted (no islet graft) KC of C57BL/6 mice (*n* = 3) was used to calculate the fold change of gene expression. After 7 days, the graft-containing kidneys were removed, processed with Trizol (Thermo Fisher Scientific, ON, Canada) and cryopreserved at −80 °C. Total RNA was extracted using the RNeasy Mini Kit (Qiagen, Mississauga, ON Canada) as per the manufacturer's protocol, followed and 1 μg of RNA was reverse transcribed into cDNA using the High Capacity cDNA Reverse Transcription Kit (Applied Biosystems- Thermo Fisher Scientific, MA, USA). All validated FAM MGB primer-probe mixes (FAM: Fluorescein amidites, MGB: minor groove binder, Applied Biosystems TaqMan Gene Expression Assay, MA, USA) were listed in Table S1. Subsequently, cDNA was amplified using the StepOnePlus™ Real-Time PCR system with StepOne software v2.3 (Applied Biosystems, MA, USA), in MicroAmp Fast Optical 96 well plates (Applied Biosystems, MA, USA), using TaqMan Fast Advanced Master Mix (Applied Biosystems, MA, USA). Quantitative gene expression values were determined by the ΔΔCT method (CT: Cycle threshold) through normalization to the housekeeping gene, β-actin, and the calibrator naïve BALB/c mouse kidney control [9,10]. Gene expression data were expressed as relative fold change to the sham transplant.

4. Islet graft immunohistochemical analysis

Formalin-fixed islets grafts were processed, embedded in paraffin block and sectioned at a thickness of 5 μm. Subsequently, grafts were stained with hematoxylin and eosin (H&E). For immunohistochemical analysis, tissue slides were rehydrated, antigen retrieved and blocked with 20% normal goat serum (NGS) (Jackson ImmunoResearch, PA,

USA) for 1 h. After blocking, sections were incubated with the respective primary antibodies of anti-guinea pig α-insulin (1:5, Agilent CA, USA), and mouse anti-glucagon (1:5000, Sigma-Aldrich, ON, Canada) for an hour, followed by 3 series of washing with 1x PBS. Subsequently, sections were incubated with respective secondary antibodies of goat anti-guinea pig Alexa fluor 488, and goat anti-mouse Alexa fluor 594 for 1 h at room temperature (1:200, Thermo Fisher Scientific, ON, Canada). After incubation, tissue sections were washed with 1xPBS, 3 times, and subsequently coverslipped with 100 µL of DAPI anti-fade reagent (Thermo Fisher Scientific, ON, Canada) and dried at room temperature in the dark. The presence of FoxP3 cells in the grafts was assessed using the avidin-biotin complex (ABC) method with peroxidase and diaminobenzidine as the chromogen. Briefly, after rehydration, antigen retrieval was performed with 0.5 M citrate buffer (pH 5.5, Thermo Fisher Scientific, ON, Canada). Subsequently, sections were blocked with 20% NGS, followed by incubated with rabbit anti-FoxP3 (1:100, Novus Biologicals, CO, USA) at 4 °C overnight. The next day, sections were washed with 1xPBS, 3 times, and subsequently, incubated with biotinylated goat anti-rabbit (Thermo Fisher Scientific, ON, Canada) for an hour. The Vectastain ABC-peroxidase kit (MJS BioLynx inc, ON, Canada) was used to amplify the signal, and diaminobenzidine (DAB; Sigma-Aldrich) was used for visualization. Sections were counterstained with H&E.

To assess immune cells infiltration in the grafts, procured grafts were preserved in cryomatrix (Epredia™ Cryomatrix™ embedding resin, Thermo Fisher Scientific, ON, Canada), immediately frozen at -20°C and tissue samples were cut in consecutive 5-µm sections on a cryotome (Leica CM3050 S Cryostat, Leica Biosystems Inc. ON, Canada), air dried, and fixed with cold acetone for 2 min. Subsequently, sections were air-dried, immersed in 1X PBS and blocked with 20% NGS (Jackson ImmunoResearch, PA, USA). Next, sections were incubated with respective primary antibodies of rat anti-mouse CD4, rat anti-mouse CD8 and rat anti-mouse CD68 (1/200, Bio-Rad, CA, USA) for 1 h, followed by incubated for an hour with goat anti-rat Alex flour 594 (1:200, Thermo Fisher Scientific, ON, Canada). Slides were washed three times with 1xPBS between the primary and secondary antibody incubations. Subsequently, sections were counter-stained with insulin (1:5, Dako, CA, USA) and mounted with DAPI anti-fade reagent (Thermo Fisher Scientific, ON, Canada). To quantify the immune cell infiltrations (CD4, CD8 and CD68), multiple sections were stained, imaged, and quantified using the ImageJ software (https://imagej.nih.gov/ij/).

5. Assessment of tolerance induction by allogeneic skin graft transplantation

Skin grafts were conducted on long-term functioning islet allograft recipients, to investigate the possibly of transplant tolerance [11,12]. A separate cohort of alloislet transplant studies were conducted where diabetic C57BL/6 mice (n = 10) received 500 BALB/c islets + 4 mg CsA microparticles + CTLA4-Ig (10 mg/kg, 0, 2, 4, and 6 posttransplant). Mice that maintained normoglycemia for 100 days received skin grafts from C57BL/6 (syngeneic grafts), BALB/c (islet donor-matched) and C3H (third-party). Naïve, non-islet transplanted and no CsA + CTLA4-Ig treated C57BL/6 mice received skin grafts similar to the experimental recipients. In brief, approximately 1 cm^2 of the donor's flank skin was transplanted at the left (BALB/c skin) and right (C3H skin) flank, and dorsally (between the flanks; C57BL/6 skin) and then secured with pressure bandages for 8 days. Subsequently, bandages were removed, and skin grafts with a ruler were digitally photographed at 8D, 10D, 12D, 14D, 16D, 18D, 20D, and 23D. In addition, skin grafts were observed every day to assess rejection. A ruler was used to calculate the area of skin remaining. We utilized digital photographs to calculate the area of skin remaining. Skin grafts were considered rejected when less than 10% of viable tissue remained (< 0.1 cm^2 tissue). The rejected and accepted skin grafts were collected and stained with H&E.

References

1. Sanchez, A.; Vilajato, J.L.; Alonso, M.J. Development of Biodegradable Microspheres and Nanospheres for the Controlled-Release of Cyclosporine-A. *Int J Pharmaceut* **1993**, *99*, 263–273, doi:Doi 10.1016/0378-5173(93)90369-Q.
2. Ankrum, J.A.; Miranda, O.R.; Ng, K.S.; Sarkar, D.; Xu, C.J.; Karp, J.M. Engineering cells with intracellular agent-loaded microparticles to control cell phenotype. *Nat Protoc* **2014**, *9*, 233–245. https://doi.org/10.1038/nprot.2014.002.
3. Kuppan, P.; Kelly, S.; Polishevska, K.; Hojanepesov, O.; Seeberger, K.; Korbutt, G.S.; Pepper, A.R. Co-localized immune protection using dexamethasone-eluting micelles in a murine islet allograft model. *Am J Transplant* **2020**, *20*, 714–725. https://doi.org/10.1111/ajt.15662.
4. Naeem, M.; Bae, J.; Oshi, M.A.; Kim, M.S.; Moon, H.R.; Lee, B.L.; Im, E.; Jung, Y.; Yoo, J.W. Colon-targeted delivery of cyclosporine A using dual-functional Eudragit((R)) FS30D/PLGA nanoparticles ameliorates murine experimental colitis. *Int J Nanomedicine* **2018**, *13*, 1225–1240. https://doi.org/10.2147/IJN.S157566.
5. He, Y.; Liu, Y.; Liu, Y.; Wang, J.; Zhang, X.; Lu, W.; Ma, Z.; Zhu, X.; Zhang, Q. Cyclosporine-loaded microspheres for treatment of uveitis: In vitro characterization and in vivo pharmacokinetic study. *Invest Ophthalmol Vis Sci* **2006**, *47*, 3983–3988. https://doi.org/10.1167/iovs.05-1373.
6. Jaiswal, J.; Gupta, S.K.; Kreuter, J. Preparation of biodegradable cyclosporine nanoparticles by high-pressure emulsification-solvent evaporation process. *J Control Release* **2004**, *96*, 169–178. https://doi.org/10.1016/j.jconrel.2004.01.017.
7. Pepper, A.R.; Gala-Lopez, B.; Pawlick, R.; Merani, S.; Kin, T.; Shapiro, A.M. A prevascularized subcutaneous device-less site for islet and cellular transplantation. *Nat Biotechnol* **2015**, *33*, 518–523. https://doi.org/10.1038/nbt.3211.
8. Hassouna, T.; Seeberger, K.L.; Salama, B.; Korbutt, G.S. Functional Maturation and In Vitro Differentiation of Neonatal Porcine Islet Grafts. *Transplantation* **2018**, *102*, e413-e423. https://doi.org/10.1097/TP.0000000000002354.
9. Zammit, N.W.; Walters, S.N.; Seeberger, K.L.; O'Connell, P.J.; Korbutt, G.S.; Grey, S.T. A20 as an immune tolerance factor can determine islet transplant outcomes. *JCI Insight* **2019**, *4*. https://doi.org/10.1172/jci.insight.131028.
10. Yamane, K.; Anazawa, T.; Tada, S.; Fujimoto, N.; Inoguchi, K.; Emoto, N.; Nagai, K.; Masui, T.; Okajima, H.; Takaori, K.; et al. Mitomycin C treatment improves pancreatic islet graft longevity in intraportal islet transplantation by suppressing proinflammatory response. *Sci Rep-Uk* **2020**, *10*. https://doi.org/10.1038/s41598-020-69009-8.
11. Lee, S.J.; Kim, H.J.; Byun, N.R.; Park, C.G. Donor-Specific Regulatory T Cell-Mediated Immune Tolerance in an Intrahepatic Murine Allogeneic Islet Transplantation Model with Short-Term Anti-CD154 mAb Single Treatment. *Cell Transplant* **2020**, *29*, 0963689720913876. https://doi.org/10.1177/0963689720913876.
12. Woodward, K.B.; Zhao, H.; Shrestha, P.; Batra, L.; Tan, M.; Grimany-Nuno, O.; Bandura-Morgan, L.; Askenasy, N.; Shirwan, H.; Yolcu, E.S. Pancreatic islets engineered with a FasL protein induce systemic tolerance at the induction phase that evolves into long-term graft-localized immune privilege. *Am J Transplant* **2020**, *20*, 1285–1295. https://doi.org/10.1111/ajt.15747.
